# Triglyceride-rich lipoprotein and LDL particle subfractions and their association with incident type 2 diabetes: the PREVEND study

**DOI:** 10.1186/s12933-021-01348-w

**Published:** 2021-07-28

**Authors:** Sara Sokooti, Jose L. Flores-Guerrero, Hiddo J. L. Heerspink, Margery A. Connelly, Stephan J. L. Bakker, Robin P. F. Dullaart

**Affiliations:** 1 Department of Internal Medicine, University Medical Center Groningen, University of Groningen, Groningen, The Netherlands; 2 Department of Clinical Pharmacy and Pharmacology, University Medical Center Groningen, University of Groningen, Groningen, The Netherlands; 3 Laboratory Corporation of America® Holdings (LabCorp), Morrisville, NC USA

**Keywords:** TRL particles, TRL size, LDL particles, LDL size, HOMA-β, HOMA-IR, Type 2 diabetes

## Abstract

**Background:**

Triglyceride-rich lipoproteins particles (TRLP) and low density lipoprotein particles (LDLP) vary in size. Their association with β-cell function is not well described. We determined associations of TRLP and LDLP subfractions with β-cell function, estimated as HOMA-β, and evaluated their associations with incident T2D in the general population.

**Methods:**

We included 4818 subjects of the Prevention of Renal and Vascular End-Stage Disease (PREVEND) study without T2D at baseline. TRLP and LDLP subfraction concentrations and their average sizes were measured using the LP4 algorithm of the Vantera nuclear magnetic resonance platform. HOMA-IR was used as measure of insulin resistance. HOMA-β was used as a proxy of β-cell function.

**Results:**

In subjects without T2D at baseline, very large TRLP, and LDL size were inversely associated with HOMA-β, whereas large TRLP were positively associated with HOMA-β when taking account of HOMA-IR. During a median follow-up of 7.3 years, 263 participants developed T2D. In multivariable-adjusted Cox regression models, higher concentrations of total, very large, large, and very small TRLP (reflecting remnants lipoproteins) and greater TRL size were associated with an increased T2D risk after adjustment for relevant covariates, including age, sex, BMI, HDL-C, HOMA-β, and HOMA-IR. On the contrary, higher concentrations of large LDLP and greater LDL size were associated with a lower risk of developing T2D.

**Conclusions:**

Specific TRL and LDL particle characteristics are associated with β-cell function taking account of HOMA-IR. Moreover, TRL and LDL particle characteristics are differently associated with incident T2D, even when taking account of HOMA-β and HOMA-IR.

**Supplementary Information:**

The online version contains supplementary material available at 10.1186/s12933-021-01348-w.

## Background

The prevalence of type 2 diabetes (T2D) is rapidly increasing worldwide with an estimated global prevalence of 548 million people in 2045 [1]. It is known for decades that T2D is featured by abnormalities in circulating lipoproteins, comprising elevations in triglyceride-rich apolipoprotein B (apoB)-containing lipoproteins and low levels of high density lipoproteins (HDL) [2–4]. Thus, T2D confers elevated levels of large sized very low-density lipoproteins (VLDL) and smaller sized low-density lipoproteins (LDL) [5, 6]. Interestingly, recent studies have suggested that abnormalities in triglyceride-rich apolipoprotein B (apoB)-containing lipoproteins may precede insulin resistance [7–12], which in turn is a major risk factor for T2D development [13–15]. Nonetheless, while exaggerated deposition of cholesterol in the pancreatic β cell is likely to adversely affect insulin secretion [16–20], little information is available on the role of circulating VLDL and LDL and their subfractions in β cell failure [16, 21].

Recently, a nuclear magnetic resonance (NMR) spectroscopy-based method was developed for quantifying triglyceride-rich lipoprotein (TRL) and LDL particle and subfraction concentrations in human plasma [22]. Using NMR methodology it has been suggested that large VLDL particles and VLDL size as well as small LDL particles may be positively associated with incident T2D. By contrast, concentrations of small VLDL and large LDL particles as well as LDL size were inversely associated with incident T2D, independent of established risk factors including glucose and insulin resistance [23–25]. Although evidence is mounting that HDL cholesterol and HDL subfractions predict incident T2D even independent of homeostasis model assessment of insulin resistance (HOMA-IR) [26], the association of TRL and LDL particle and subfraction concentrations with β-cell function, estimated as homeostasis model assessment of β-cell function (HOMA-β) have not been determined in a population-based cohort so far.

The aims of the present study were (i) to cross-sectionally explore the associations between TRL and LDL particle and subfraction concentrations with HOMA β taking account of insulin resistance and (ii) to longitudinally determine the associations of TRL and LDL particle and subfraction concentrations with incident T2D. To this end NMR-derived lipoproteins we measured in the population-based Prevention of Renal and Vascular End-Stage Disease (PREVEND) study.

## Methods

### Study design and participants

The study was performed among participants of the Prevention of REnal and Vascular ENd-stage Disease (PREVEND) cohort study. Details of the study design and recruitment have been described elsewhere [27]. Briefly, all residents of Groningen aged 28 to 75 years were invited to participate in this study from 1997 to 1998. Pregnant women, individuals with type 1 diabetes and T2D using insulin were excluded. Participants with a urinary Albumin excretion UAE ≥ 10 mg/L (n = 6000) and control group with a UAE < 10 mg/L (n = 2592), a total of 8592 individuals, completed an extensive first screening. A second screening round took place from 2001 to 2003, encompassing 6893 participants and was considered the “baseline” for the current study. Individuals with missing NMR (n = 136) or covariate data (n = 909) at baseline and those with missing data on diabetes and follow-up (n = 722) were excluded, leaving 308 subjects with pre-existing diabetes and 4,818 subjects without diabetes at baseline of the present study (Additional file 1: Figure S1). Baseline lipoprotein characteristics of 722 subjects who lost the follow-up were not different from those who could be followed longitudinally except for lower medium LDLP and higher small LDLP in subjects who were lost to follow-up (Additional file 1: Table S1).

The protocol for the PREVEND study was approved by the local ethics committee of the University Medical Center Groningen (approval number: MEC96/01/022). All subjects included in the present analysis provided written informed consent to participate, and the study procedures were conducted according to the Declaration of Helsinki.

### Clinical and laboratory measurement

During two outpatient clinic visits separated by 3 weeks, baseline data on demographics, lifestyle parameters, cardiovascular and renal disease history, smoking habits and medication use were collected. During two outpatient clinic visits separated by 3 weeks, baseline data on demographics, lifestyle parameters, cardiovascular and renal disease history, smoking habits and medication use were collected. Smoking and alcohol use were based on self-reports. Self-reported alcohol consumption was recorded in as: absence (no alcohol consumption), 1 to 4 units/month, 2 to 7 units/week, 1 to 3 units/day, or > 3 units/day; 1 unit was considered to contain 10 g of alcohol [28]. We categorized alcohol consumption as high intake > 3 units/day vs. the rest (no high intake ≤ 3 units/day. Information on medication use was combined with information from IADB.nl, a database containing information on prescribed medication in public pharmacies in The Netherlands since 1999 (http://www.iadb.nl/). Height and weight were measured with the participants standing without shoes or heavy outer garments. Body mass index (BMI) was calculated by dividing weight in kilograms by height in meters squared. Systolic and diastolic blood pressure values were recorded as the means of the last 2 recordings of the second visit.

Plasma samples were taken from participants after an overnight fast, were prepared by centrifugation at 4 °C and stored at − 80 °C until analysis. Fasting plasma glucose (FPG) was measured by dry chemistry (Eastman Kodak, Rochester, NY, USA). EDTA-anticoagulated plasma samples were stored at − 80 °C. Insulin was measured with an immunoturbidometric assay (Diazyme Laboratories, Poway, CA, USA). HOMA-IR was calculated as fasting plasma insulin (mU/L) × fasting plasma glucose (mmol/L)/22.5. HOMA-β was calculated 20 × fasting plasma insulin (mU/L)/[fasting plasma glucose (mmol/L) − 3.5] [29]. High sensitivity C-reactive protein (hs-CRP) was assayed by nephelometry (Dade Behring Diagnostic, Marburg, Germany). Urinary albumin was measured by nephelometry (Dade Behring Diagnostic, Marburg, Germany). Serum creatinine was determined by Kodak Ektachem dry chemistry (Eastman Kodak, Rochester, New York) and serum cystatin C level by nephelometry (BN II N) (Dade Behring Diagnostic, Marburg, Germany). Estimated glomerular filtration rate (eGFR) was estimated using the Chronic Kidney Disease Epidemiology Collaboration (CKD-EPI) combined creatinine-cystatin C equation.

Lipoprotein parameters were measured by *NMR LipoProfile*® testing at Labcorp (Morrisville, NC). NMR spectra were collected on an optimized version of the NMR LipoProfile test (LP4 algorithm) [22, 26, 30]*.* Very large, large, medium, small, and very small triglyceride rich lipoprotein particles (TRLP) and large, medium, and small LDL particles (LDLP) were quantified using the conventional deconvolution method and the amplitudes of their spectroscopically distinct lipid methyl group NMR signals [31]. Total TRLP were calculated as the sum of the concentrations of very large, large, medium, small, and very small TRLP. Total LDLP were calculated as the sum of the concentrations of large, medium, and small LDLP. Mean TRL and LDL size were calculated using the weighted averages derived from the sum of the diameters of each subfraction. Estimated ranges of particle diameter for the TRL and LDL subfractions were as follows: very large TRLP, 90–240 nm; large TRLP, 50–89 nm; medium TRLP, 37–49 nm; small TRLP, 30–36 nm; very small TRLP, 24–29 nm; large LDLP, 21.5–23 nm; medium LDLP, 20.5–21.4 nm; and small LDLP, 19–20.4 nm. Total cholesterol (TC), triglycerides, HDL-cholesterol (HDL-C), LDL-cholesterol (LDL-C), and apoB using equations derived from lipoprotein measures assayed on a Vantera Clinical NMR Analyzer platform using Partial Least-Squares (PLS) regression models. Non-HDL cholesterol was calculated as the difference between TC and HDL-C.

### Outcome ascertainment

Follow-up time was defined as the period between the baseline measurement (second screening) and the date of ascertainment of T2D. In case a person moved to an unknown destination, the census date was the date of removal from the municipal registry. T2D was ascertained if one or more of the following criteria were met: (1) FPG ≥ 7.0 mmol/L (126 mg/dL), (2) random sample plasma glucose ≥ 11.1 mmol/L (200 mg/dL), (3) self-report of a physician diagnosis of T2D, and (4) initiation of glucose-lowering medication use, retrieved from a central pharmacy registry.

### Statistical analyses

All analyses were conducted using the statistical packages IBM SPSS (version 24.0.1; SPSS, Chicago, IL, USA) and STATA/SE (version 14; StataCorp, College Station, TX, USA). For all analyses, 2-sided *P* values < 0.05 were considered statistically significant. Results were expressed as mean ± standard deviation (SD), median with interquartile range (IQR) or number (percentage) for normally distributed, skewed and categorical data, respectively. Baseline characteristics are presented for the whole cohort and according to T2D status (pre-existing T2D at baseline, developed T2D and did not develop T2D during follow-up time). Differences in baseline characteristics between the 3 groups were tested using one-way ANOVA or Kruskal Wallis tests for skewed continuous variables with subsequent Bonferroni correction, and *X*^*2*^ tests for categorical variables. Univariate linear regression analyses were performed to assess the cross-sectional associations of TC, non-HDL cholesterol, LDL-C, HDL-C, triglycerides, apoB, TRL and LDL subfractions and size with HOMA-IR and HOMA-β in the total population including subjects with and without diabetes at baseline. Subsequently, P-values for interaction, indicating difference between each variable and T2D status at baseline in unadjusted analysis, were calculated. Then, uni- and multivariable linear regression analyses were performed in subjects without T2D and with T2D at baseline, separately. Multivariable linear regression models were adjusted for sex, age, and HOMA-IR or HOMA-β; plus BMI, lipid-lowering medication, anti-hypertensive medication (model 1b) for subjects without T2D; plus glucose-lowering medication for subjects with pre-existing T2D (model 1a). Variables with a skewed distribution were log_e_ transformed. Crude and multivariable Cox proportional hazards regression analyses were performed to examine the associations between each predictor and incident T2D. In addition, hazards ratios (HR) with 95% confidence intervals (CIs) were calculated per 1 SD increment of predictors (log_e_ transformed for variables which were not normally distributed). The following five cumulative models were used for adjustment: age and sex (models 1); plus alcohol use, BMI, family history of diabetes, lipid-lowering medication and anti-hypertensive medication (models 2); plus HDL-C (models 3a) or HDL size (models 3b); plus HOMA-β (models 4a and 4b); plus HOMA-IR (models 5a and 5b). Interactions were tested to assess statistical evidence for effect modification by using statins or high alcohol use in crude analyses. For predictors of which the interaction term was significant, we performed subgroup analyses.

## Results

### Baseline characteristics

308 individuals had pre-existing T2D at baseline, 263 subjects developed T2D during follow-up of 7.3 (6.1–7.7) years and 4,555 subjects did not develop T2D during follow-up (Table 1). Individuals who developed T2D were more likely to be men and to have a family history of diabetes more frequently than participants who did not develop T2D. Subjects with pre-existing T2D were older and consumed less alcohol in comparison with two other groups without T2D at baseline. Individuals with pre-existing T2D and who developed T2D had a higher BMI, Systolic blood pressure (SBP), FPG, HOMA-IR, hs-CRP but lower eGFR, and they used anti-hypertensive and lipid-lowering medication more frequently than individuals who did not develop T2D. HOMA-β was lower in subjects with pre-existing T2D. While TC was significantly higher in subjects who developed T2D compared to subjects with pre-existing T2D, non-HDL cholesterol was significantly higher in those who developed T2D compared to the subjects those who did not develop T2D. Triglycerides, apoB, total TRLP, TRLP subfractions and size (except small TRLP), as well as total LDLP and small LDLP were higher, whereas HDL-C, medium and large LDLP and LDL size were lower in individuals with pre-existing T2D and those who developed T2D compared to participants who did not develop T2D.

**Table 1:** Baseline characteristics of subjects with pre-existing diabetes, who developed and who did not develop T2D

Variables	Diabetes at baseline	Incident diabetes		*P* value
		Yes	No	
Participants, n	308	263	4555	
General characteristics				
Female, %	43.8	38.0	51.2	< 0.001^a,b,c^
Age, year	62.6 ± 9.9	57.0 ± 9.7	52.5 ± 11.6	< 0.001^a,b,c^
Lifestyle parameters				
Current smoker, %	20.1	28.1	26.8	0.626
Alcohol consumption,				0.082
None, %	39.8	26.7	23.0	0.032
1–4 units per month, %	15.1	14.5	17.4	
2–7 units per week, %	24.3	28.2	32.4	
1–3 units per day, %	17.1	23.7	23.1	
> 3 units per day, %	3.6	6.8	4.1	
Family history of diabetes %	31.5	33.1	16.6	< 0.001^b,c^
Body composition				
Weight, kg	87.7 ± 16.5	88.6 ± 15.3	78.7 ± 14.0	< 0.001^a,b^
Height, cm	171.1 ± 10.0	172.5 ± 9.2	173.2 ± 9.4	0.001^a^
BMI, kg/m^2^	29.9 ± 5.2	29.8 ± 4.7	26.2 ± 4.0	< 0.001^a,b^
Waist circumference, cm	101.9 ± 12.7	101.7 ± 12.6	90.6 ± 12.1	< 0.001^a,b^
Blood pressure				
Systolic blood pressure, mmHg	135.3 ± 19.8	135.9 ± 19.7	124.3 ± 17.7	< 0.001^a,b^
Diastolic blood pressure, mmHg	75.3 ± 9.0	77.3 ± 9.0	72.9 ± 8.9	< 0.001^a,b,c^
Hypertension, %	63.6	55.5	25.8	< 0.001^a,b,c^
Anti-hypertensive medication, %	44.8	35.1	14.0	< 0.001^a,b,c^
Lipid-lowering medication, %	26.3	19.0	7.3	< 0.001^a,b,c^
Statin use, %	23.8	16.1	6.6	< 0.001^a,b,c^
Glucose-lowering medication, %	56.2	N/A	N/A	
Metformin use, %	23.7	N/A	N/A	
Sulfonylurea, %	40.5	N/A	N/A	
Insulin, %	1.3	N/A	N/A	
Glucose homeostasis				
FPG, mmol/L	8.1 ± 2.4	5.7 ± 0.7	4.8 ± 0.6	< 0.001^a,b,c^
Insulin, mU/L	14.3 (9.5–21.4)	13.6(9.2–20.3)	7.7 (5.6–11.3)	< 0.001^a,b^
HOMA-IR, (mU/L^2^)/22.5	4.9 (3.1–7.9)	3.4 (2.3–5.4)	1.6 (1.1–2.4)	< 0.001^a,b,c^
HOMA‐β, %	68.7 (42.1–113.6)	131.1 (84.4–202.2)	133.3 (91.7–203.3)	< 0.001^a,c^
Hs-CRP, mg/L	2.6 (1.3–4.9)	2.1 (1.1–3.8)	1.2 (0.5–2.8)	< 0.001^a,b^
Renal function				
eGFR, mL/min per 1.73 m^2^	85.9 (73.0–98.9)	90.0 (79.0–100.5)	95.0 (83.0–105.0)	< 0.001^a,b,c^
Urinary Albumin excretion, mg/24 h	16.3 (8.2–46.0)	12.2 (7.8–30.0)	8.4 (6.0–14.4)	< 0.001^a,b,c^
Lipids and lipoproteins				
Total cholesterol, mg/dL	190.6 ± 40.2	198.5 ± 36.2	193.5 ± 34.4	0.023^c^
Non-HDL cholesterol, mg/dL	146.0 ± 38.4	152.4 ± 35.6	141.5 ± 35.7	< 0.001^b^
LDL-C, mg/dL	112.7 ± 32.6	118.2 ± 30.0	113.9 ± 29.1	0.050
HDL-C, mg/dL	44.6 ± 9.9	45.9 ± 9.9	52.0 ± 12.2	< 0.001^a,b^
Triglycerides (total), mg/dL	127.7 (88.7–179.1)	134.6 (89.2–198.9)	91.0 (65.1–133.9)	< 0.001^a,b^
ApoB, mg/dL	93.9 ± 24.7	97.6 ± 23.2	90.2 ± 23.1	< 0.001^a,b^
TRLP, nmol/L	164.0 (126.1–212.0)	176.9 (132.3–220.0)	147.4 (109.2–189.7)	< 0.001^a,b^
Very large TRLP, nmol/L	0.06 (0.01–0.20)	0.06 (0.01–0.27)	0.04 (0.01–0.09)	< 0.001^a,b^
Large TRLP, nmol/L	4.8 (1.9–9.3)	5.5 (2.4–10.8)	1.7 (0.2–5.0)	< 0.001^a,b^
Medium TRLP, nmol/L	17.3 (7.5–31.7)	18.2 (8.0–32.2)	11.3 (5.4–21.3)	< 0.001^a,b^
Small TRLP, nmol/L	48.5 (22.4–84.3)	47.3 (24.3–84.3)	46.8 (25.2–79.4)	0.073
Very small TRLP, nmol/L	77.5 (40.0–118.5)	86.8 (44.4–130.8)	68.8 (37.2–108.0)	0.006^b^
TRL size, nm	50.0 ± 9.2	51.3 ± 9.9	45.5 ± 8.2	< 0.001^a,b^
LDLP, nmol/L	1567.9 (1252.7–1833.0)	1611.6 (1343.7–1895.6)	1467.3 (1226.1–1727.3)	< 0.001^a,b^
Large LDLP, nmol/L	198.1 (55.2–373.7)	244.2 (54.4–414.5)	378.9 (197.0–567.1)	< 0.001^a,b^
Medium LDLP, nmol/L	300.5 (39.8–677.4)	362.6 (72.8–755.7)	386.6 (126.3–723.0)	< 0.001^a,b^
Small LDLP, nmol/L	843.2 (508.1–1214.7)	821.1 (519.2–1194.7)	533.8 (305.9–838.6)	< 0.001^a,b^
LDL size, nm	20.7 ± 0.6	20.8 ± 0.6	21.1 ± 0.5	< 0.001^a,b^

Data are the mean ± SD, median (interquartile range) unless otherwise indicated. Significance was tested by one-way ANOVA tests and Kruskal Wallis tests where appropriate

*BMI* Body mass index, *hs-CRP* high sensitivity C-reactive protein, *FPG* fasting plasma glucose, *HOMA-IR* Homeostatic model assessment of insulin resistance, *HOMA‐β* Homeostatic model assessment of β-cell function, *eGFR* estimated glomerular filtration rate, *LDL-C* Low-density lipoprotein cholesterol, *HDL-C* High-density lipoprotein cholesterol, *ApoB* apolipoprotein B, *LDLP* low density lipoprotein particles, *TRLP* triglyceride rich lipoprotein particles, *N/A* not applicable

^a^Different between the group with T2D at baseline and the group without T2D at follow up

^b^Different between the group with incident T2D and the group without T2D at follow up

^c^Different between the group with T2D at baseline and the group with incident T2D at follow up at *p* value < 0.05 (by Bonferroni correction)

In subjects (1) with pre-existing T2D, (2) those who developed T2D and (3) those who did not develop T2D during follow-up, lipids and lipoprotein values were compared between participants using a statin and those who did not (Additional file 1: Table S2). Overall, TC, non-HDL-C, LDL-C, apoB, small TRLP, total LDLP, large and medium LDLP and LDL size were lower in statin users compared to non-statin users in each group.

Furthermore, lipids and lipoprotein values were compared between participants who consumed high alcohol and those who did not consume high alcohol in subjects with pre-existing T2D, those who developed T2D and those did not develop T2D during follow-up (Additional file 1: Table S3). TC, non-HDL-C, LDL-C, triglycerides, apoB, total, very large, large and very small TRLP, TRL size, and small LDLP where higher, whereas small TRLP and medium LDLP where lower in subjects with high alcohol intake compared to subject who did not consume high amounts of alcohol particularly in those subjects who did not develop T2D during follow-up.

### Cross-sectional associations

Univariable and multivariable linear regression analyses were performed to show the association between lipid and lipoprotein measures with HOMA-IR (Table 2) and HOMA-β (Table 3). In the total population, TC, LDL-C, TRLP, very large and large TRLP and TRL size, as well as LDLP were positively, whereas HDL-C, medium LDLP and LDL size were inversely associated with HOMA-IR in the univariate model. Non-HDL-C, apoB, were positively associated with HOMA-IR in the multivariable model adjusted for age, sex, HOMA-β, BMI and medication use in the group without T2D at baseline but not in the group with T2D at baseline (Table 2). Triglycerides, medium TRLP and small LDLP were positively associated with HOMA-IR, whereas large LDLP were inversely associated with HOMA-IR in the multivariable model in subjects with and without T2D at baseline.

**Table 2:** Uni- and multivariable linear regression analyses between lipids, TRL and LDL particle concentrations, subfractions and sizes with HOMA-IR

	Total population (n = 5124), std β, P-values	P-value for interaction	T2D at baseline (n = 308), std β, P-values		No T2D at baseline (n = 4818), std β, P-values	
Variables	Univariate		Univariate	Model 1a	Univariate	Model 1b
Total cholesterol	0.091***	0.233	0.052	− 0.005	0.112***	0.067***
Non-HDL cholesterol	0.213***	**0.022**	0.099	0.065	0.229***	0.143***
LDL-C	0.127***	0.055	0.046	0.036	0.149***	0.083***
HDL-C	− 0.370***	0.087	− 0.173**	− 0.267***	− 0.357***	− 0.229***
Triglycerides	0.411***	**0.040**	0.274***	0.303***	0.402***	0.279***
ApoB	0.231***	**0.012**	0.102	0.072	0.246***	0.153***
TRLP	0.237***	0.055	0.090	0.105	0.237***	0.143***
Very large TRLP	0.249***	0.612	0.267***	0.284***	0.228***	0.214***
Large TRLP	0.399***	0.459	0.329***	0.330***	0.385***	0.266***
Medium TRLP	0.249***	**0.001**	0.055	0.137*	0.254***	0.159***
Small TRLP	− 0.013	0.301	− 0.058	− 0.082	− 0.004	0.008
Very small TRLP	0.018	0.336	0.066	0.147*	0.015	− 0.020
TRL size	0.331***	0.403	0.349***	0.345***	0.315***	0.208***
LDLP	0.227***	0.186	0.158**	0.131*	0.238***	0.142***
Large LDLP	− 0.278***	**0.014**	− 0.197***	− 0.258***	− 0.252***	− 0.195***
Medium LDLP	− 0.068***	0.918	− 0.056	− 0.106	− 0.047***	− 0.035
Small LDLP	0.159 ***	**0.002**	0.243***	0.297***	0.135***	0.086***
LDL size	− 0.302***	0.404	− 0.232***	− 0.357***	− 0.271***	− 0.195***

Standardized regression coefficients are shown (Stdß) are shown. Log_e_ transformed values are used for variables with a skewed distribution

Model 1a: sex, age, and HOMA‐β, BMI, lipid-lowering medication, anti-hypertensive medication, and glucose-lowering medication

Model 1b: sex, age, and HOMA‐β, BMI, lipid-lowering medication, anti-hypertensive medication

*HOMA-IR* Homeostatic model assessment of insulin resistance, *HOMA‐β* Homeostatic model assessment of β-cell function, *BMI* Body mass index, *LDL-C* Low-density lipoprotein cholesterol, *ApoB* apolipoprotein B, *LDLP* low density lipoprotein particles, *TRLP* triglyceride rich lipoprotein particles

^*^P < 0.05; **P < 0.01; ***P < 0.001. P-value for interaction indicates difference between each variable and T2D status at baseline in unadjusted analysis. Bold values indicate statistically significant.

**Table 3:** Uni- and multivariable linear regression analyses between lipids, TRL and LDL particle concentrations, subfractions and sizes with HOMA‐β

	Total population (n = 5124, std β, P-values	P-value for interaction	T2D at baseline ((n = 308), std β, P-values		No T2D at baseline (n = 4818), std β, P-values	
Variables	Univariate		Univariate	Model 1a	Univariate	Model 1b
Total cholesterol	0.028*	**0.014**	− 0.081	− 0.027	0.034*	0.001
Non-HDL cholesterol	0.067***	**0.011**	− 0.046	− 0.014	0.086***	0.021
LDL-C	0.044**	0.067	− 0.041	− 0.038	0.050*	0.009
HDL-C	− 0.118***	0.201	− 0.150**	− 0.055	− 0.156***	− 0.058***
Triglycerides	0.134***	**0.011**	0.030	− 0.084	0.183***	0.057***
ApoB	0.072***	**0.010**	− 0.042	− 0.011	0.093***	0.025
TRLP	0.065***	0.077	− 0.018	− 0.030	0.091***	0.027
Very large TRLP	0.026	**0.003**	− 0.069	− 0.197**	0.063***	− 0.038*
Large TRLP	0.132***	**0.020**	0.014	− 0.136*	0.180***	0.049**
Medium TRLP	0.105***	0.054	0.012	0.016	0.135***	0.067***
Small TRLP	− 0.003	0.977	− 0.004	0.028	− 0.007	0.007
Very small TRLP	0.011	0.899	0.005	− 0.005	0.012	0.015
TRL size	0.106***	0.116	0.047	− 0.082	0.149***	0.033*
LDLP	0.069***	**0.025**	− 0.030	− 0.020	0.088***	0.023
Large LDLP	− 0.070***	0.130	− 0.052	0.005	− 0.112***	− 0.030*
Medium LDLP	0.001	0.343	0.027	0.079	− 0.018	0.012
Small LDLP	0.045**	0.475	0.005	− 0.106	0.070***	0.022
LDL size	− 0.068***	**0.031**	− 0.003	0.121*	− 0.116***	− 0.036*

Standardized regression coefficients are shown (Stdß) are shown. Log_e_ transformed values are used for variables with a skewed distribution

Model 1a: sex, age, and HOMA‐β, BMI, lipid-lowering medication, anti-hypertensive medication, and glucose-lowering medication

Model 1b: sex, age, and HOMA‐β, BMI, lipid-lowering medication, anti-hypertensive medication

*HOMA-IR* Homeostatic model assessment of insulin resistance, *HOMA‐β* Homeostatic model assessment of β-cell function, *BMI* Body mass index, *LDL-C* Low-density lipoprotein cholesterol, *ApoB* apolipoprotein B, *LDLP* low density lipoprotein particles, *TRLP* triglyceride rich lipoprotein particles

^*^P < 0.05; **P < 0.01; ***P < 0.001. P-value for interaction indicates difference between each variable and status of T2D at baseline in unadjusted analysis. Bold values indicate statistically significant.

In the total population, total and medium TRLP, as well as small LDLP were positively, whereas HDL-C, medium TRLP, and large LDLP were inversely associated with HOMA-β in the univariate model. TC, non HDL-C, triglycerides, apoB, very large and large TRLP and LDLP were positively associated with HOMA-β in the univariate model in the group without T2D at baseline but not in the group with T2D at baseline. Moreover, in the multivariable linear regression analyses very large TRLP were inversely associated with HOMA-β both in participants with and without T2D at baseline. Large TRLP were inversely associated with HOMA-β in the group with T2D at baseline but positively associated with HOMA-β in the group without T2D. On the other hand, LDL size was positively associated with HOMA-β in the group with T2D at baseline but inversely associated with HOMA-β in the group without T2D at baseline (Table 3).

### Associations of HOMA-IR and HOMA-β with incident T2D

First, Cox proportional hazard regression analyses were performed for HOMA-IR and HOMA-β (Additional file 1: Table S4). Higher HOMA-IR was associated with a higher risk of T2D in crude analysis and after adjustment for relevant covariates. The association between HOMA-IR and incident T2D remained significant after further adjustment for HOMA-β and HDL-C or HDL size [HR per 1 SD increase 5.92 (95% CI 4.76–7.36), 5.79 (95% CI 4.65–7.22), respectively]. Oppositely, higher HOMA-β was associated with a lower risk of T2D and the association between HOMA-β and incident T2D remained significant after further adjustment for HOMA-IR and HDL-C or HDL size [HR per 1 SD increase 0.21 (95% CI 0.17–0.26), 0.20 (95% CI 0.16–0.26), respectively].

### Associations of non-HDL cholesterol, LDL-C, triglycerides, and apoB with incident T2D

In addition, Cox proportional hazard regression analyses were performed for non-HDL cholesterol, LDL-C, triglycerides, and apoB and incident T2D (Additional file 1: Table S5). In crude analyses, higher concentrations of non-HDL cholesterol, LDL-C, triglycerides, and apoB were associated with an increased risk of T2D. After adjustment for relevant covariates including age and sex (models 1), high alcohol intake, BMI, family history of diabetes, anti-hypertensive and lipid-lowering medication (models 2), HDL-C and HDL size (models 3a and 3b), HOMA-β (models 4a and 4b), and HOMA-IR (models 5a and 5b), the plasma triglyceride concentration was the only variable that remained significantly associated with incident T2D. The HR per 1 SD increase in the two last models was 1.29 (95% CI 1.13–1.47), 1.23 (95% CI 1.07–1.42), respectively.

### Associations of TRL and LDL particle subfractions and size with incident T2D

Cox proportional hazard regression analyses were performed for TRL and LDL particle subfractions and size with incident T2D (Additional file 1: Tables S6 and S7, respectively).

**Fig. 1:**
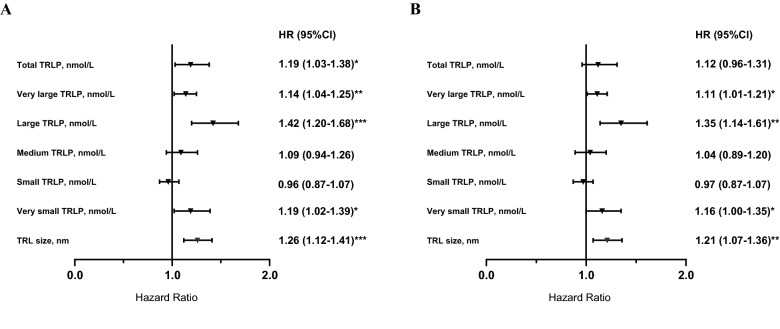
Association between TRLP subfractions and risk of T2D in 4818 people without diabetes at baseline. Multivariable hazard ratios (95% confidence intervals) for risk of T2D are expressed per increase of TRLP subfractions. Hazard ratios (95 CIs) were derived from Cox proportional hazards regression models adjusted for sex, age, high alcohol intake, BMI, lipid-lowering medication, anti-hypertensive medication, family history of diabetes, HOMA- β, HOMA-IR, and HDL-C, in **A**, or HDL size in **B**. *P < 0.05; **P < 0.01; ***P < 0.001. *LDLP* low density lipoprotein particles, *HOMA-IR* Homeostatic model assessment of insulin resistance, *HOMA‐β* Homeostatic model assessment of β-cell function, *BMI* Body mass index, *HDL-C* High-density lipoprotein cholesterol

Among TRL particle subfractions, higher total TRLP, very large, large, very small TRLP, and a larger TRLP size were associated with increased risk of T2D, whereas small TRLP were associated with a lower risk of T2D in crude analysis. The HR per 1 SD increase for very large TRLP in the two last models was 1.14 (95% CI 1.04–1.25), 1.11 (95% CI 1.01–1.21), respectively. The HR per 1 SD increase for large TRLP in the two final models was 1.42 (95% CI 1.20–1.68) and 1.35 (95% CI 1.14–1.61), respectively. Furthermore, the HR per 1 SD increase for very small TRLP in the two final models was 1.19 (95% CI 1.02–1.39), 1.16 (95% CI 1.00–1.35), respectively. The HR per 1 SD increase for TRL size in the two final models were 1.26 (95% CI 1.12–1.41) and 1.21 (95% CI 1.07–1.36), respectively (Fig. 1).

**Fig. 2:**
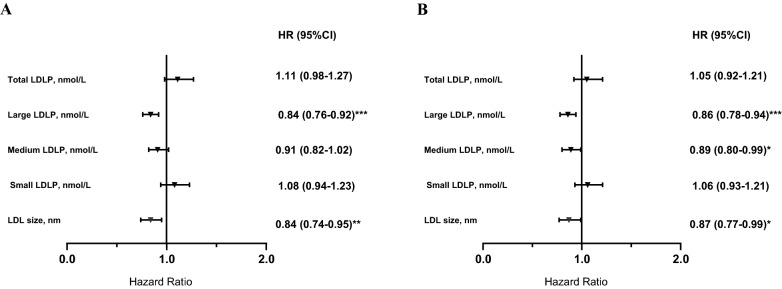
Association between LDL subfractions and risk of T2D in 4818 people without diabetes at baseline. Multivariable hazard ratios (95% confidence intervals) for risk of T2D are expressed per increase of LDL subfractions. Hazard ratios (95 CIs) were derived from Cox proportional hazards regression models adjusted for sex, age, high alcohol intake, BMI, lipid-lowering medication, anti-hypertensive medication, family history of diabetes, HOMA- β, HOMA-IR, and HDL-C, in **A**, or HDL size in **B**. *P < 0.05; **P < 0.01; ***P < 0.001. *LDLP* low density lipoprotein particles, *HOMA-IR* Homeostatic model assessment of insulin resistance, *HOMA‐β* Homeostatic model assessment of β-cell function, *BMI* Body mass index, *HDL-C* High-density lipoprotein cholesterol

Among LDL particle subfractions, higher concentrations of total and small LDLP, whereas lower concentration of large and medium LDLP as well as smaller LDL size were associated with increased risk of T2D in crude analyses. The association remained significant for large and medium LDLP as well as LDL size, after adjustment for age and sex (model 1), high alcohol intake, BMI, and family anti-hypertensive and lipid-lowering medication (model 2), HDL-C or HDL size (model 3a and 3b), HOMA-β (model 4a and 4b). In the two last models adjusted for HOMA-IR (model 5a and 5b), the HR per 1 SD increase for large LDLP was 0.84 (95% CI 0.76–0.92), 0.86 (95% CI 0.78–0.94), respectively and the HR per 1 SD increase for LDL size was 0.84 (95% CI 0.74–0.95), 0.87 (95% CI 0.77–0.99), respectively. The association remained significant for medium LDLP in model 5b [HR per 1 SD increase 0.89 (95% CI 0.80–0.99)] (Fig. 2).

### Secondary analyses TRL and LDL particle subfractions and size with incident T2D in various subgroups

**Fig. 3:**
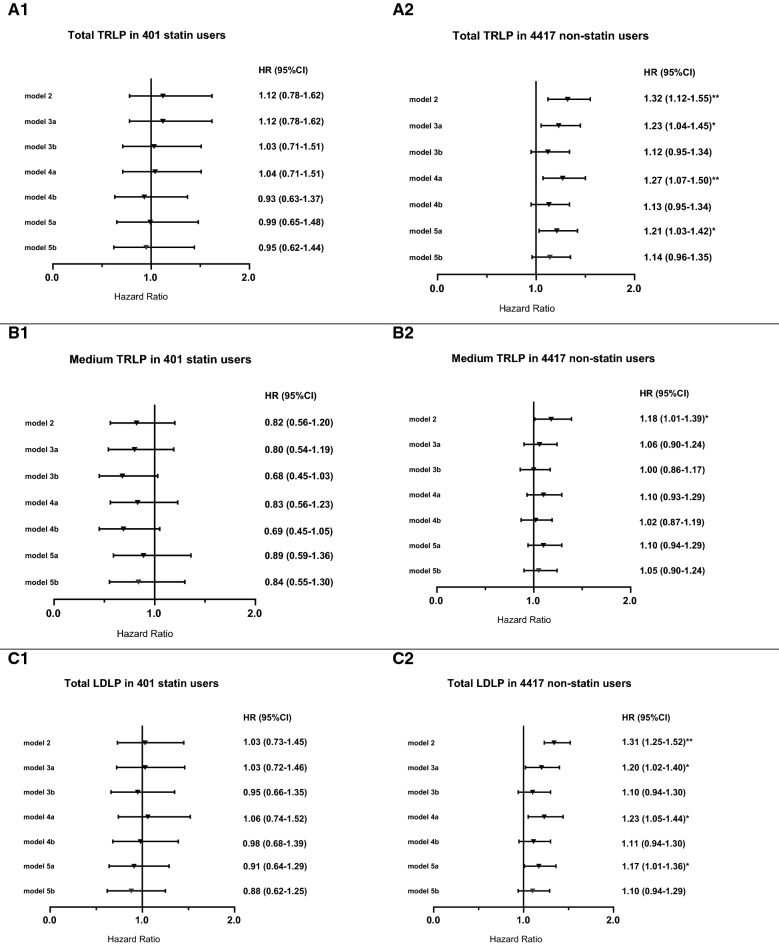
Association between total TRLP, medium TRLP, total LDLP and risk of T2D in 401 statin users and 4417 non users. **A1** total TRLP in statin users; **A2** total TRLP in non-statin users; **B1** medium TRLP in statin users; **B2** medium TRLP in non-statin users; **C1** total LDLP in statin users; **C2** total LDLP in non-statin users. *P < 0.05; **P < 0.01; ***P < 0.001. Model 2: model 1 ++ high alcohol intake, BMI, lipid-lowering medication, anti-hypertensive medication, and family history of diabetes. Model 3a: model 2 + HDL-C; Model 3b: model 2 + HDL size. Model 4a: model 3a + HOMA‐β; Model 4b: model 3b + HOMA‐β. Model 5a: model 4a + HOMA‐ IR; Model 5b: model 4b + HOMA‐IR. *TRLP* triglyceride rich lipoprotein particles, *HOMA-IR* Homeostatic model assessment of insulin resistance, *HOMA‐β* Homeostatic model assessment of β-cell function, *BMI* Body mass index, *HDL-C* High-density lipoprotein cholesterol

To find potential effect modifications, we tested for interactions by using statins (yes vs. no) and high alcohol intake (> 3 vs. ≤ 3 units per day). In crude analyses, we found significant effect modification for using statins with total TRLP (*P* = 0.042), medium TRLP (*P* = 0.030), and total LDLP (*P* = 0.001). Consequently, secondary analyses were performed among subgroups of individuals who used statins and who did not use statins (Fig. 3). Higher concentrations of total TRLP and LDLP were associated with increased risk of T2D development after adjustment for age and sex, high alcohol intake, BMI, anti-hypertensive and family history of diabetes (model 2), HDL-C (model 3a), HOMA-β (model 4a), and HOMA-IR (model 5a) in non-statin users but not statin users (Fig. 3).

In addition, an effect modification for high alcohol intake was found for large TRLP (*P* = 0.042), TRL size (*P* = 0.034), and small LDLP (*P* = 0.012). Higher levels of large TRLP, and TRL size were associated with increased risk of T2D development after adjustment for all covariates in individuals without high alcohol intake but not in subjects with high alcohol consumption (Additional file 1: Table S8).

## Discussion

In the current large population-based cohort study, we first investigated the association between TRL and LDL particle concentrations, determined with a novel NMR-based algorithm, with HOMA-β. In subjects without T2D at baseline, very large TRLP, large LDLP and LDL size were inversely associated with HOMA-β, whereas large and medium TRLP and TRL size were positively associated with HOMA-β when taking account of HOMA-IR, BMI and medication use. Second, during a median follow-up of 7.3 years, very large, large and very small TRLP and TRL size were positively associated, whereas large LDLP and LDL size were inversely associated, with incident T2D after adjustment for multiple T2D risk factors, including BMI, HDL-C, or alternatively HDL size, HOMA-β, and HOMA-IR. In secondary analyses, higher concentrations of total TRLP and LDLP were particularly associated with a higher risk of incident T2D in non-statin users. In addition, higher levels of large TRLP, and TRL size were associated with an increased risk of T2D in participants who did not consume high amounts of alcohol.

It is well appreciated that insulin resistance plays a key role in the development of lipoprotein abnormalities featured by elevated triglycerides, higher large VLDLP and increased VLDL size, as well as a shift from larger towards smaller LDLP and decreased LDL size [7–12]. Circulating concentrations of triglyceride rich apoB-containing lipoproteins, which vary in size from large VLDL1 (50–80 nm) to smaller VLDL2 (30–50 nm), are differently affected in the context of high triglyceride and glucose levels. Subjects with elevated plasma triglyceride levels overproduce VLDL1 due to high fat content in the liver and a failure of insulin to suppress VLDL1 synthesis [32]. Additionally in patients with diabetes, raised plasma triglyceride levels are due to increased production of VLDL1, contributing to relatively long-lived remnants and small LDL. However, with optimal triglyceride levels few remnants are produced and mostly large LDL particles are formed [33]. Using a novel NMR platform-derived algorithm which captures five different TRL subfractions and three LDL subfractions we found that in subjects with pre-existing diabetes and subjects who developed T2D, total TRLP, TRLP subfractions and size (except small TRLP), as well as total LDLP and small LDLP were higher, whereas large LDLP, medium LDLP and LDL size were lower compared to participants who did not develop T2D. Relationships of these TRL and LDL characteristics with HOMA-IR were confirmed both individuals with and without T2D at baseline. In addition, a modest, but hitherto unappreciated, positive relationship of HOMA-β with TRL size and an inverse relationship with LDL size was observed independent of HOMA-IR, particularly in subjects without T2D.

NMR-measured very large and large TRLP and TRLP size were positively associated with incident T2D. Regarding the associations of various TRL subfractions and size with incident T2D, the current results are generally consistent with prior studies [23, 25, 34]. Notably, very small TRLP (24–29 nn), which should be considered remnant particles as they correspond in size to intermediate density lipoproteins (23–27 nm, LP3 algorithm) [35], were associated with incident T2D as well. These findings point to a potential role of remnant particles in diabetes development besides the well appreciated causal atherogenic potential of the cholesterol content in remnant particles [36]. For LDL particles, higher concentration of large LDL and greater LDL size were suggested to be inversely associated, whereas higher concentrations of small LDL were positively associated with incident T2D in previous studies [9, 23, 25]. Although we found that LDL size and large LDL particles were inversely associated with risk of T2D development, we did not find a strong association between small LDL particles and incident T2D. Hence, a smaller LDL size may be associated more robustly with T2D development [9, 10, 23, 25, 37] than absolute concentrations of small LDLP.

In comparison with our NMR findings, of conventional (apo)lipoprotein and lipid measures, the plasma triglyceride concentration was the only variable that was associated with incident T2D in multivariable analysis in the whole study population at risk. Despite apoB being a causal factor in atherosclerosis development [38], apoB was not associated with incident T2D in adjusted analyses, consistent with the presently observed lack of association with the LDL particle concentration. Taken together, the present findings highlight the relevance of lipoprotein subfraction measurement in assessing the association of lipoprotein characteristics with the risk of T2D development.

Remarkably, the associations of TRL and LDL particle characteristics with incident T2D as documented in our study were independent of, and only modestly diminished after, adjustment for HOMA-IR despite the presently reiterated strong association of HOMA-IR with T2D development [present study] [39]. Moreover, such associations were independent of HOMA-β. In the interpretation of these results, it should be recognized that HOMA-IR is a measure of insulin resistance on glucose metabolism and hence does not necessarily represent a strong proxy of insulin resistance on free fatty acid metabolism in adipose and liver tissue [40–42]. Furthermore, HOMA-β is a static measure of insulin secretion with only a modest relationship with dynamic tests of insulin secretory capacity by pancreatic β cells [29, 43, 44].

Evidence is mounting that cholesterol accumulation in pancreatic ß-cells may give rise to ß-cell dysfunction [17, 18, 45, 46]. Of further interest, VLDL and LDL particles are able to modulate ß-cell function [16], possibly in part attributable to pancreatic steatosis [47]. While oxidized LDL induces ß-cell apoptosis [48], both LDL and VLDL particles may decrease ß-cell proliferation by reducing cyclin B1 expression [49]. Furthermore, addition of human LDL to cultured islets impairs glucose-stimulated insulin secretion, mediated by the LDL receptor [50]. Interestingly, proprotein convertase subtilisin/kexin type 9 (PCSK9) deficiency results in increased accumulation of cholesteryl esters in pancreatic islets, coinciding with increased intracellular insulin but decreased circulating insulin [17]. In humans, circulating PCSK9 is associated with intermediate density lipoproteins [35], and high PCSK9 plasma concentrations may associate with increased risk of T2D development [18]. Although, it was recently found that PCSK9 inhibition does not appear to significantly impact on insulin secretion in mice and humans [51, 52], two other studies suggested that human PCSK9 loss-of-function variants were associated with a raised risk to develop new-onset diabetes whereas PCSK9 may mediate 11% of insulin resistance in obese and depressed patients [53, 54]. Thus, the mechanisms responsible for the association of specific TRL and LDL particle characteristics with T2D development are still unprecisely known and should await further studies.

Reasoning that associations of TRL and LDL particles characteristics with incident T2D could in part be dependent on cholesterol accumulation in pancreatic β-cells [45, 46], and that inhibition of 3-hydroxy-3-methylglutaryl-CoA reductase could affect glucose tolerance and insulin secretion [55–57], secondary analyses were performed according to statin use. Statin users had lower levels of TC, LDL-C, non-HDL-C, apoB [58] and a slightly lower LDL particle size [59] as expected. In agreement with the possibility that exposure to higher circulating levels of lipoprotein-associated cholesterol would increase diabetes risk, higher total TRLP and LDLP concentrations were associated with incident T2D in crude and in fully adjusted analysis in non-statin users, but not in statin users. In comparison, using NMR spectroscopy (LP3 algorithm), Mackey et al. could not find a potential effect modification by use of statin on the association of NMR- measured lipoprotein particles with incident T2D [25].

Of further relevance, alcohol consumption has been included in risk scores for T2D prediction [60–62]. Although low and moderate alcohol consumption was found to be inversely associated with T2D development risk [63–66], high alcohol intake is associated with metabolic disorders including glucose, lipid and lipoprotein abnormalities, which may contribute to pancreatic toxicity [67–70]. Alcohol-induced hypertriglyceridemia is due to increased VLDL secretion, impaired lipolysis and increased free fatty acid fluxes from adipose tissue to the liver [68]. As expected, LDL-C, triglycerides, TRL subfractions, TRL size, and small LDLP were higher in subjects with high alcohol intake compared to the subjects who did not consume high amounts of alcohol [58]. Nonetheless, large TRLP and a greater TRL size were not associated with incident T2D in individuals with high alcohol consumption.

Several methodological considerations of our study need to be considered. HOMA-β and HOMA-IR values are intricately interdependent estimates of β-cell function and insulin resistance on glucose metabolism, respectively [29]. This underscores our approach to take account of HOMA-IR when evaluating HOMA-β because insulin secretion will adapt to increased demands due to insulin resistance [43, 71, 72]. Furthermore, HDL particles are able to affect β-cell function [20, 73], and HDL-C makes part of established diabetic risk models [74, 75]. Recently, HDL size was found to be associated with incident T2D even independent of HDL-C [26]. In the current study, we therefore adjusted HDL-C or alternatively for HDL size. Notably, statin use is associated with a modestly increased risk of incident T2D [76–78]. This adverse effect on glucose regulation does not outweigh the cardiovascular benefit of such treatment [79] but requires adjustment for statin use when evaluating the association of lipoprotein subfractions on incident T2D.

The current study has several strengths and limitations. A strength of this study is that it includes a large number of participants with a large age range, and the long-term follow-up, from the general population. Given the design of our longitudinal observation study, cause-effect relationships cannot be ascertained with certainty. Although the majority of the PREVEND participants were of north European descent, our findings are in line with previous studies which were performed in the population with different ethnicities [24, 25]. Moreover, alcohol intake was collected by participants’ self-report, making that the possibility of underestimation of alcohol consumption cannot be excluded.

## Conclusions

In conclusion, TRL and LDL particle characteristics are differentially associated with incident T2D. Very large, large, and very small TRLP and TRL size were positively associated, whereas large LDLP and LDL size were inversely associated with incident T2D. Such associations were particularly found in non-statin users for total TRLP, and among participants who did not take high amounts of alcohol for large TRLP and TRL size.

## Supplementary Information

**Additional file 1: Figure S1.** Detailed information on reason of exclusion and numbers of missing values of NMR measurement ,other covariates, and loss to follow-up for the analyses on TRL and LDL Particle subfractions and their association with incident T2D in The PREVEND study. **Table S1.** Baseline characteristics of 722 subjects lost to the follow up and 4818 subjects who completed the follow up. **Table S2.** Lipids and lipoproteins characteristics of subjects with pre-existing diabetes at baseline, subjects who developed T2D and subjects who did not develop T2D in 401 statin users and 4417 non users. **Table S3.** Lipids and lipoproteins characteristics of subjects with pre-existing diabetes at baseline, subjects who developed T2D and subjects who did not develop T2D in 214 subjects with high alcohol intake and 4912 subjects with no high alcohol intake. **Table S4.** Association between HOMA‐ β, HOMA-IR and risk of T2D in 4818 people without diabetes at baseline. **Table S5.** Association between non-HDL cholesterol, LDL-C, triglycerides, and apoB and risk of T2D in 4818 people without diabetes at baseline. **Table S6.** Association between TRLP subfractions and risk of T2D in 4818 people without diabetes at baseline. **Table S7.** Association between LDL subfractions and risk of T2D in 4818 people without diabetes at baseline. **Table S8.** Association between large TRLP, TRL size , and small LDLP and risk of T2D in 203 subjects with high alcohol intake and 4615 subjects with no high alcohol intake.

## Data Availability

The datasets used and analysed during the current study are available from the corresponding author on reasonable request.

## Abbreviations

apoB: Apolipoprotein B
BMI: Body mass index
CKD-EPI: Chronic Kidney Disease Epidemiology Collaboration
eGFR: Estimated glomerular filtration rate
FPG: Fasting plasma glucose
HOMA-IR: Homeostasis model assessment of insulin resistance
HOMA-β: Homeostasis model assessment of β-cell function
hs-CRP: High sensitivity C-reactive protein
HDL-C: High density lipoprotein cholesterol
LDLP: Low density lipoprotein particles
LDL-C: LDL-cholesterol
NMR: Nuclear magnetic resonance
PREVEND: Prevention of Renal and Vascular End-Stage Disease
PLS: Partial Least-Squares
PCSK9: Proprotein convertase subtilisin/kexin type 9
T2D: Type 2 diabetes
TRLP: Triglyceride-rich lipoproteins particles
TC: Total cholesterol
UAE: Urinary albumin excretion
VLDL: Very low-density lipoproteins
