# Supplementary material for: Triglyceride-rich lipoprotein and LDL particle subfractions and their association with incident type 2 diabetes: the PREVEND study
Source: Cardiovasc Diabetol. 2021 Jul 28;20:156. doi: 10.1186/s12933-021-01348-w (PMC8320057; doi:10.1186/s12933-021-01348-w)
Supplement: Supplementary file 1 — Additional file 1: Figure S1. Detailed information on reason of exclusion and numbers of missing values of NMR measurement ,other covariates, and loss to follow-up for the analyses on TRL and LDL Particle subfractions and their association with incident T2D in The PREVEND study. Table S1. Baseline characteristics of 722 subjects lost to the follow up and 4818 subjects who completed the follow up. Table S2. Lipids and lipoproteins characteristics of subjects with pre-existing diabetes at baseline, subjects who developed T2D and subjects who did not develop T2D in 401 statin users and 4417 non users. Table S3. Lipids and lipoproteins characteristics of subjects with pre-existing diabetes at baseline, subjects who developed T2D and subjects who did not develop T2D in 214 subjects with high alcohol intake and 4912 subjects with no high alcohol intake. Table S4. Association between HOMA‐ β, HOMA-IR and risk of T2D in 4818 people without diabetes at baseline. Table S5. Association between non-HDL cholesterol, LDL-C, triglycerides, and apoB and risk of T2D in 4818 people without diabetes at baseline. Table S6. Association between TRLP subfractions and risk of T2D in 4818 people without diabetes at baseline. Table S7. Association between LDL subfractions and risk of T2D in 4818 people without diabetes at baseline. Table S8. Association between large TRLP, TRL size , and small LDLP and risk of T2D in 203 subjects with high alcohol intake and 4615 subjects with no high alcohol intake. [file 12933_2021_1348_MOESM1_ESM.docx]

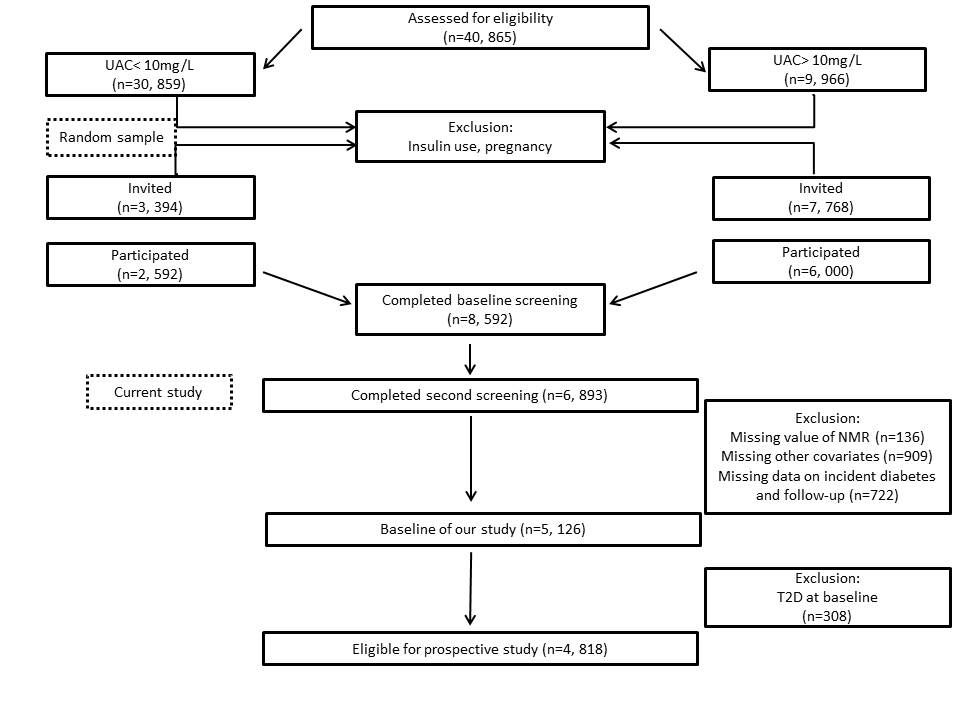


**Figure S1**. Detailed information on reason of exclusion and numbers of missing values of NMR measurement ,other covariates, and loss to follow-up for the analyses on TRL and LDL Particle subfractions and their association with incident T2D in The PREVEND study.

| **Table S1**.Baseline characteristics of 722 subjects lost to the follow up and 4818 subjects who completed the follow up | | | | | | |
| --- | --- | --- | --- | --- | --- | --- |
| Variables | | Lost follow-up | | Completed follow-up | *P* value | |
| Participants, n | | 722 | | 4818 |  | |
| Female, % | | 55.5 | | 50.5 | 0.011 | |
| Age, year | | 56.2±13.9 | | 52.8±11.6 | <0.001 | |
| Lipids and lipoproteins | |  | |  |  | |
| Total cholesterol, mg/dL | | 195.5±35.5 | | 193.8±34.5 | 0.221 | |
| Non-HDL cholesterol, mg/dL | | 143.7±36.3 | | 142.1±35.8 | 0.266 | |
| LDL-C, mg/dL | | 115.5±29.8 | | 114.1±29.2 | 0.227 | |
| HDL-C, mg/dL | | 51.8±13.4 | | 51.7±12.1 | 0.815 | |
| Triglycerides (total), mg/dL | | 92.4 (64.7-135.8) | | 92.5 (65.8-137.3) | 0.767 | |
| ApoB, mg/dL | | 91.6±23.5 | | 90.6±23.2 | 0.286 | |
| TRLP, nmol/L | | 147.6(109.1-188.2) | | 148.5 (110.0-192.4) | 0.479 | |
| Very large TRLP, nmol/L | | 0.04 (0.01-0.09) | | 0.04 (0.01-0.09) | 0.520 | |
| Large TRLP, nmol/L | | 2.0 (0.2-5.5) | | 1.93 (0.2-5.3) | 0.392 | |
| Medium TRLP, nmol/L | | 11.9 (5.3-22.7) | | 11.6 (5.5-22.1) | 0.947 | |
| Small TRLP, nmol/L | | 46.2 (25.2-79.1) | | 46.8 (25.1-79.5) | 0.941 | |
| Very small TRLP, nmol/L | | 67.2 (36.4-106.7) | | 69.7 (37.5-109.4) | 0.363 | |
| TRL size, nm | | 46.0±8.8 | | 45.8±8.4 | 0.546 | |
| LDLP, nmol/L | | 1514.7 (1264.4-1764.4) | | 1475.9 (1231.0-1737.1) | 0.061 | |
| Large LDLP, nmol/L | | 389.3 (199.1-570.5) | | 371.2 (188.8-562.3) | 0.387 | |
| Medium LDLP, nmol/L | | 339.1 (96.9-674.5) | | 386.3 (124.2-724.1) | 0.041 | |
| Small LDLP, nmol/L | | 601.1 (339.3-933.4) | | 544.3 (311.5-862.4) | 0.007 | |
| LDL size, nm | | 21.0±0.5 | | 21.1±0.5 | 0.438 | |
| Data are the mean ± SD, median (interquartile range) unless otherwise indicated. Significance was tested by t-tests and Wilcoxon tests for normal distribution and skewed distribution of continuous values respectively. LDL-C: Low-density lipoprotein cholesterol; HDL-C: High-density lipoprotein cholesterol; ApoB: apolipoprotein B; LDLP: low density lipoprotein particles; TRLP: triglyceride rich lipoprotein particles. | | | | | | |
|  |  | |  | | |  |

| **Table S2**. Lipids and lipoproteins characteristics of subjects with pre-existing diabetes at baseline, subjects who developed T2D and subjects who did not develop T2D in 401 statin users and 4417 non users | | | | | | | |
| --- | --- | --- | --- | --- | --- | --- | --- |
|  | | Diabetes at baseline | Incident diabetes | | |  | |
| Variables | |  | Yes | | No | *P* value | |
| **Statin users** | | 74 | | 46 | 355 | |  |
| Total cholesterol, mg/dL | | 160.6±33.0* | | 177.0±38.6* | 180.3±30.1* | | <0.001^a c^ |
| Non-HDL cholesterol, mg/dL | | 114.3±30.3* | | 129.3±39.1* | 130.7±28.8* | | <0.001^a^ |
| LDL-C, mg/dL | | 88.7±24.5* | | 97.8±32.7* | 101.9±24.2* | | <0.001^a^ |
| HDL-C, mg/dL | | 42.9±8.9 | | 47.7±9.1 | 49.6±10.6* | | <0.001^a^ |
| Triglycerides (total), mg/dL | | 116.8 (90.6-167.0) | | 134.9 (78.3-193.4) | 106.9 (79.2-150.6)* | | 0.098 |
| ApoB, mg/dL | | 76.9±20.2* | | 82.8±25.7* | 84.2±18.9* | | 0.020 ^a^ |
| TRLP, nmol/L | | 133.0 (99.9-165.9)* | | 155.9 (113.4-213.4) | 153.5 (118.9-184.9) | | 0.250 |
| Very large TRLP, nmol/L | | 0.07 (0.01-0.20) | | 0.09 (0.02-0.52) | 0.05 (0.01-0.12)* | | 0.002 ^b^ |
| Large TRLP, nmol/L | | 4.8 (2.2-9.8) | | 6.2 (2.6-10.7) | 3.0 (0.6-7.5)* | | <0.001^a b^ |
| Medium TRLP, nmol/L | | 17.2 (9.9-28.1) | | 16.6 (8.1-30.6) | 17.1 (8.3-26.1)* | | 0.965 |
| Small TRLP, nmol/L | | 35.4 (19.0-65.6)* | | 34.3 (14.5-52.6)* | 43.4 (25.5-69.6)* | | 0.310 |
| Very small TRLP, nmol/L | | 63.8 (29.5-98.8)* | | 93.3 (50.9-129.0) | 76.4 (44.2-110.0) | | 0.013 ^c^ |
| TRL size, nm | | 51.0±8.0 | | 54.2±10.9 | 47.7±8.4* | | <0.001 ^a b^ |
| LDLP, nmol/L | | 1252.9 (1088.2-1493.4)* | | 1312.9 (1072.8-1684.4)* | 1408.6 (1198.1-1601.4)* | | 0.045 ^a^ |
| Large LDLP, nmol/L | | 109.5 (29.8-212.3)* | | 114.4 (15.5-270.9)* | 236.1 (100.5-382.8)* | | <0.001 ^a b^ |
| Medium LDLP, nmol/L | | 137.0 (0.1-408.0)* | | 243.1 (34.3-529.2)* | 209.2 (26.6-467.5)* | | 0.369 |
| Small LDLP, nmol/L | | 897.5 (684.7-1152.2) | | 838.6 (622.9-1102.4) | 798.3 (535.4-1092.3)* | | 0.066 |
| LDL size, nm | | 20.5±0.5* | | 20.6±0.5* | 20.8±0.5* | | <0.001 ^a^ |
|  | |  | |  |  | |  |
| **Non-users** | | 234 | | 217 | 4200 | |  |
| Total cholesterol, mg/dL | | 199.9±37.7 | | 202.0±34.5 | 194.6±34.5 | | 0.001 ^b^ |
| Non-HDL cholesterol, mg/dL | | 154.8±36.4 | | 156.5±33.3 | 142.3±36.1 | | <0.001^a b^ |
| LDL-C, mg/dL | | 119.7±31.4 | | 121.9±28.2 | 114.8±29.2 | | <0.001 ^a b^ |
| HDL-C, mg/dL | | 45.2±10.2 | | 45.5±10.0 | 52.3±12.3 | | <0.001 ^a b^ |
| Triglycerides (total), mg/dL | | 133.0 (88.1-194.9) | | 131.9 (92.9-201.9) | 90.1(64.3-132.6) | | <0.001^a b^ |
| ApoB, mg/dL | | 99.3±23.5 | | 100.2±21.6 | 90.7±23.4 | | <0.001^a b^ |
| TRLP, nmol/L | | 179.6 (136.3-221.2) | | 180.9 (132.4-220.5) | 147.0 (107.9-189.8) | | <0.001 ^a b^ |
| Very large TRLP, nmol/L | | 0.05 (0.01-0.20) | | 0.06 (0.01-0.26) | 0.04 (0.01-0.09) | | <0.001 ^a b^ |
| Large TRLP, nmol/L | | 4.8 (1.9-9.3) | | 5.3 (2.4-10.9) | 1.7 (0.2-4.9) | | <0.001^a b^ |
| Medium TRLP, nmol/L | | 17.8 (7.2-33.0) | | 17.8 (7.9-32.9) | 10.9 (5.3-20.9) | | <0.001 ^a b^ |
| Small TRLP, nmol/L | | 53.2 (22.9-96.2) | | 50.1 (24.8-85.7) | 47.2(25.1-80.3) | | 0.156 |
| Very small TRLP, nmol/L | | 85.9 (45.1-126.3) | | 88.0 (44.1-131.3) | 68.6 (36.8-108.0) | | 0.005 ^b^ |
| TRL size, nm | | 49.8±9.6 | | 50.8±9.7 | 45.3±8.2 | | <0.001 ^a b^ |
| LDLP, nmol/L | | 1611.9 (1353.6-1906.9) | | 1646.3 (1402.9-1939.2) | 1471.1 (1229.9-1737.6) | | <0.001 ^a b^ |
| Large LDLP, nmol/L | | 246.8 (77.8-426.4) | | 260.6 (61.8-436.5) | 390.0(208.5-576.4) | | <0.001 ^a b^ |
| Medium LDLP, nmol/L | | 357.5 (79.6-733.1) | | 413.3 (74.2-825.6) | 401.6 (132.4-736.1) | | 0.001 ^a b^ |
| Small LDLP, nmol/L | | 770.8 (420.9-1257.9) | | 800.5 (463.6-1223.0) | 520.6 (295.8-809.5) | | <0.001 ^a b^ |
| LDL size, nm | | 20.8±0.6 | | 20.8±0.6 | 21.1±0.5 | | <0.001 ^a b^ |
| Data are the mean ± SD, median (interquartile range) unless otherwise indicated. Significance was tested by one-way ANOVA tests and Kruskal Wallis tests where appropriate. ^a^Difference between the group with T2D at baseline and the group without T2D at follow up. ^b^Difference between the group with incident T2D and the group without T2D at follow up. ^c^Difference between the group with T2D at baseline and the group with incident T2D at follow up at *p* value<0.05 (by Bonferroni correction). * Difference between the groups using a statin and the group who did not use s statin at *p* value<0.05. LDL-C: Low-density lipoprotein cholesterol; HDL-C: High-density lipoprotein cholesterol; ApoB: apolipoprotein B; LDLP: low density lipoprotein particles; TRLP: triglyceride rich lipoprotein particles. | | | | | | | |
|  |  | | | | | | |

| **Table S3**. Lipids and lipoproteins characteristics of subjects with pre-existing diabetes at baseline, subjects who developed T2D and subjects who did not develop T2D in 214 subjects with high alcohol intake and 4912 subjects with no high alcohol intake | | | | | | |
| --- | --- | --- | --- | --- | --- | --- |
|  | | Diabetes at baseline | Incident diabetes | |  | |
| Variables | |  | Yes | No | *P* value | |
| **High alcohol intake** | | 11 | 18 | 185 | |  |
| Total cholesterol, mg/dL | | 199.0±43.5 | 196.9±34.6 | 206.3±38.1* | | 0.533 |
| Non-HDL cholesterol, mg/dL | | 152.6±46.6 | 150.0±33.1 | 151.7±40.5* | | 0.982 |
| LDL-C, mg/dL | | 118.7±37.2 | 118.2±29.7 | 121.6±32.9* | | 0.886 |
| HDL-C, mg/dL | | 46.3±7.5 | 46.8±9.5 | 49.6±10.6* | | 0.010 ^b^ |
| Triglycerides (total), mg/dL | | 149.9 (75.8-209.5) | 107.5 (79.8-193.1) | 102.2 (70.8-170.0)* | | 0.498 |
| ApoB, mg/dL | | 98.1±31.9 | 95.1±21.6 | 96.0±26.3* | | 0.954 |
| TRLP, nmol/L | | 206.5(110.2-223.7) | 180.0 (130.4-213.6) | 160.1 (114.2-203.8)* | | 0.414 |
| Very large TRLP, nmol/L | | 0.10 (0.01-0.44) | 0.06 (0.03-0.54) | 0.06 (0.01-0.20)* | | 0.765 |
| Large TRLP, nmol/L | | 5.6 (1.9-15.0) | 4.1 (1.6-11.4) | 3.11 (1.4-7.9)* | | 0.310 |
| Medium TRLP, nmol/L | | 27.3 (3.9-33.8) | 7.5 (3.6-28.6) | 9.5 (3.4-24.1) | | 0.529 |
| Small TRLP, nmol/L | | 19.6 (6.3-61.7) | 30.2 (19.2-70.9) | 37.7 (16.1-71.2)* | | 0.752 |
| Very small TRLP, nmol/L | | 100.8 (60.6-141.4) | 103.3 (49.4-153.4) | 87.4 (51.5-134.3)* | | 0.597 |
| TRLP size, nm | | 53.4±7.7 | 52.0±10.3 | 50.4±10.1* | | 0.525 |
| LDLP, nmol/L | | 1644.8 (1170.8-2149.5) | 1611.1 (1262.1-1868.1) | 1549.8 (1280.4-1888.7)* | | 0.954 |
| Large LDLP, nmol/L | | 252.9 (0.1-326.7) | 207.8 (116.3-493.7) | 393.2 (157.9-602.8) | | 0.013 ^a^ |
| Medium LDLP, nmol/L | | 251.2 (0.1-974.4) | 318.5 (33.3-892.5) | 299.7 (57.5-699.9)* | | 0.980 |
| Small LDLP, nmol/L | | 848.3 (506.4-1500.2) | 724.5 (391.5-964.0) | 586.9 (377.9-1043.2)* | | 0.710 |
| LDL size, nm | | 20.7±0.5 | 20.9±0.5 | 21.1±0.6 | | 0.298 |
|  | |  |  |  | |  |
| **No high alcohol intake** | | 297 | 245 | 4370 | |  |
| Total cholesterol, mg/dL | | 190.3±40.1 | 198.3±36.3 | 193.0±34.1 | | 0.023^c^ |
| Non-HDL cholesterol, mg/dL | | 145.8±38.1 | 152.5±35.9 | 142.3±36.1 | | <0.001^b^ |
| LDL-C, mg/dL | | 112.4±32.4 | 118.2±30.2 | 113.6±28.9 | | 0.041^b^ |
| HDL-C, mg/dL | | 44.5±10.0 | 45.8±10.0 | 52.0±12.1 | | <0.001 ^a b^ |
| Triglycerides (total), mg/dL | | 126.2 (89.5-177.9) | 134.9 (91.9-198.9) | 90.6(64.9-133.2) | | <0.001^a b^ |
| ApoB, mg/dL | | 93.8±24.5 | 97.8±23.3 | 90.0±23.0 | | <0.001^a b^ |
| TRLP, nmol/L | | 163.9 (126.1-209.5) | 176.2 (132.2-220.0) | 146.5 (108.9-188.9) | | <0.001 ^a b^ |
| Very large TRLP, nmol/L | | 0.06 (0.01-0.20) | 0.06 (0.01-0.26) | 0.04 (0.01-0.09) | | <0.001 ^a b^ |
| Large TRLP, nmol/L | | 4.8 (1.9-9.2) | 5.6 (2.5-10.8) | 1.6 (0.2-4.9) | | <0.001^a b^ |
| Medium TRLP, nmol/L | | 17.4 (7.8-31.6) | 18.9 (8.8-32.3) | 11.4 (5.5-21.2) | | <0.001 ^a b^ |
| Small TRLP, nmol/L | | 49.3 (22.9-84.9) | 47.7 (24.5-84.9) | 47.0(25.7-79.7) | | 0.722 |
| Very small TRLP, nmol/L | | 76.1 (39.4-117.7) | 86.6 (44.3-128.7) | 67.9 (36.9-106.7) | | 0.010 ^b^ |
| TRLP size, nm | | 49.9±9.2 | 51.2±9.9 | 45.3±8.1 | | <0.001 ^a b^ |
| LDLP, nmol/L | | 1565.7 (1253.6-1832.2) | 1611.6 (1344.0-1909.8) | 1461.6 (1224.8-1720.6) | | <0.001 ^a b^ |
| Large LDLP, nmol/L | | 195.2 (55.4-379.9) | 245.3 (52.2-408.4) | 378.4(199.4-565.1) | | <0.001 ^a b^ |
| Medium LDLP, nmol/L | | 301.0 (40.3-675.9) | 362.6 (74.1-755.0) | 390.6 (128.4-723.2) | | 0.001 ^a b^ |
| Small LDLP, nmol/L | | 841.4 (506.6-1214.1) | 838.6 (519.4-1219.0) | 530.1 (303.1-832.4) | | <0.001 ^a b^ |
| LDL size, nm | | 20.7±0.6 | 20.8±0.6 | 21.1±0.5 | | <0.001 ^a b^ |
| Data are the mean ± SD, median (interquartile range) unless otherwise indicated. Significance was tested by one-way ANOVA tests and Kruskal Wallis tests where appropriate. ^a^Difference between the group with T2D at baseline and the group without T2D at follow up. ^b^Difference between the group with incident T2D and the group without T2D at follow up. ^c^Difference between the group with T2D at baseline and the group with incident T2D at follow up at *p* value<0.05 (by Bonferroni correction). * Difference between the groups with high alcohol intake and the group with no high alcohol intake at *p* value<0.05. LDL-C: Low-density lipoprotein cholesterol; HDL-C: High-density lipoprotein cholesterol; ApoB: apolipoprotein B; LDLP: low density lipoprotein particles; TRLP: triglyceride rich lipoprotein particles. | | | | | | |
|  |  | | | | | |

| **Table S4**. Association between HOMA‐ β, HOMA-IR and risk of T2D in 4818 people without diabetes at baseline | | | | | |
| --- | --- | --- | --- | --- | --- |
| HR (95% CI) per SD-increase,  *P*-values | | | | | |
|  |  | HOMA-IR |  | HOMA‐ β |  |
| Crude |  | **3.11 (2.77-3.50)***** |  | **0.87 (0.76-0.99)*** |  |
| Model 1 |  | **2.95 (2.61-3.24)***** |  | 0.87 (0.77-1.00) |  |
| Model 2 |  | **2.43 (2.09-2.81)***** |  | **0.67 (0.58-0.78)***** |  |
| Model 3a |  | **2.36 (2.03-2.75)***** |  | **0.62 (0.53-0.73)***** |  |
| Model 3b |  | **2.31 (1.98-2.70)***** |  | **0.59 (0.51-0.69)***** |  |
| Model 4a |  | **5.92 (4.76-7.36)***** |  | **0.21 (0.17-0.26)***** |  |
| Model 4b |  | **5.79 (4.65-7.22)***** |  | **0.20 (0.16-0.26)***** |  |
| HRs (95% CIs) were derived from Cox proportional hazard models.  Model 1: sex and age  Model 2: model 1+ high alcohol intake, BMI, lipid-lowering medication , anti-hypertensive medication , and family history of diabetes  Model 3a: model 2+ HDL-C; Model 3b: model 2+ HDL size  Model 4a: model 3a + HOMA-IR or HOMA‐ β; Model 4b: model 3b + HOMA-IR or HOMA‐ β  *P<0.05; **P<0.01; ***P< 0.001.  HOMA-IR: Homeostatic model assessment of insulin resistance; HOMA‐β : Homeostatic model assessment of β-cell function; BMI: Body mass index; HDL-C: High-density lipoprotein cholesterol. | | | | | |

| **Table S5**. Association between non-HDL cholesterol, LDL-C, triglycerides, and apoB and risk of T2D in 4818 people without diabetes at baseline | | | | | |
| --- | --- | --- | --- | --- | --- |
| HR (95% CI) per SD-increase,  *P*-values | | | | | |
|  |  | Non-HDL cholesterol | LDL-C | Triglycerides | ApoB |
| Crude |  | **1.35 (1.20-1.52)***** | **1.16 (1.03-1.31)*** | **1.83 (1.64-2.04)***** | **1.36 (1.21-1.53)***** |
| Model 1 |  | **1.27 (1.12-1.43)***** | 1.09 (0.97-1.24) | **1.80 (1.60-2.02)***** | **1.27 (1.13-1.43)***** |
| Model 2 |  | **1.21 (1.07-1.37)**** | 1.10 (0.97-1.24) | **1.53 (1.34-1.73)***** | **1.21 (1.07-1.36)**** |
| Model 3a |  | **1.15 (1.01-1.31)*** | 1.06 (0.93-1.19) | **1.45 (1.26-1.66)***** | 1.13 (0.99-1.28) |
| Model 3b |  | 1.07 (0.94-1.22) | 1.00 (0.88-1.13) | **1.35 (1.17-1.57)***** | 1.04 (0.91-1.19) |
| Model 4a |  | **1.17 (1.03-1.33)*** | 1.06 (0.94-1.21) | **1.53 (1.34-1.75)***** | **1.15 (1.01-1.31)*** |
| Model 4b |  | 1.07 (0.93-1.22) | 1.00 (0.88-1.13) | **1.41 (1.22-1.63)***** | 1.04 (0.91-1.19) |
| Model 5a |  | 1.11 (0.98-1.26) | 1.03 (0.91-1.16) | **1.29 (1.13-1.47)***** | 1.09 (0.97-1.24) |
| Model 5b |  | 1.06 (0.93-1.20) | 0.99 (0.88-1.12) | **1.23 (1.07-1.42)**** | 1.04 (0.91-1.18) |
| HRs (95% CIs) were derived from Cox proportional hazard models.  Model 1: sex and age  Model 2: model 1+ high alcohol intake, BMI, lipid-lowering medication , anti-hypertensive medication, and family history of diabetes  Model 3a: model 2+ HDL-C; Model 3b: model 2+ HDL size  Model 4a: model 3a + HOMA‐β; Model 4b: model 3b + HOMA‐β  Model 5a: model 4a + HOMA‐ IR; Model 5b: model 4b + HOMA‐ IR  *P<0.05; **P<0.01; ***P< 0.001.  HOMA-IR: Homeostatic model assessment of insulin resistance; HOMA‐β : Homeostatic model assessment of β-cell function; BMI: Body mass index; LDL-C: Low-density lipoprotein cholesterol; ApoB: apolipoprotein B; HDL-C: High-density lipoprotein cholesterol. | | | | | |

| **Table S6**. Association between TRLP subfractions and risk of T2D in 4818 people without diabetes at baseline | | | | | | | |
| --- | --- | --- | --- | --- | --- | --- | --- |
| HR (95% CI) per SD-increase,  *P*-values | | | | | | | |
| TRLP subfractions | Total TRLP | Very large TRLP | Large TRLP | Medium TRLP | Small TRLP | Very small TRLP | TRL size |
| Crude | **1.56 (1.36-1.80)***** | **1.46 (1.35-1.59)***** | **2.28 (1.95-2.67)***** | **1.50 (1.29-1.74)***** | **0.88 (0.79-0.98)*** | **1.34 (1.13-1.59)**** | **1.78 (1.60-1.98)***** |
| Model 1 | **1.44 (1.25-1.67)***** | **1.46 (1.35-1.58)***** | **2.16 (1.84-2.54)***** | **1.40 (1.21-1.63)***** | **0.87(0.78-0.96)**** | **1.28 (1.09-1.51)**** | **1.75 (1.57-1.95)***** |
| Model 2 | **1.30 (1.12-1.51)***** | **1.33 (1.22-1.45)***** | **1.71 (1.45-2.01)***** | 1.14 (0.99-1.32) | 0.93(0.84-1.03) | **1.24 (1.05-1.45)**** | **1.45 (1.30-1.61)***** |
| Model 3a | **1.22 (1.05-1.42)**** | **1.32 (1.22-1.44)***** | **1.62(1.37-1.90)***** | 1.04 (0.90-1.20) | 0.93(0.84-1.03) | **1.22 (1.05-1.43)*** | **1.42 (1.28-1.58)***** |
| Model 3b | 1.12 (0.96-1.31) | **1.27 (1.16-1.38)***** | **1.51 (1.27-1.80)***** | 0.97 (0.84-1.12) | 0.94(0.85-1.04) | **1.18 (1.01-1.37)*** | **1.35 (1.21-1.51)***** |
| Model 4a | **1.26 (1.08-1.46)**** | **1.36 (1.25-1.47)***** | **1.78 (1.50-2.10)***** | 1.08 (0.93-1.25) | 0.92(0.83-1.02) | **1.23 (1.05-1.44)*** | **1.50 (1.35-1.67)***** |
| Model 4b | 1.12 (0.96-1.31) | **1.29 (1.19-1.40)***** | **1.63 (1.37-1.94)***** | 0.98 (0.85-1.14) | 0.93(0.84-1.03) | **1.17 (1.01-1.36)*** | **1.41 (1.26-1.57)***** |
| Model 5a | **1.19 (1.03-1.38)*** | **1.14 (1.04-1.25)**** | **1.42 (1.20-1.68)***** | 1.09 (0.94-1.26) | 0.96 (0.87-1.07) | **1.19 (1.02-1.39)*** | **1.26 (1.12-1.41)***** |
| Model 5b | 1.12 (0.96-1.31) | **1.11 (1.01-1.21)*** | **1.35 (1.14-1.61)**** | 1.04 (0.89-1.20) | 0.97 (0.87-1.07) | **1.16 (1.00-1.35)*** | **1.21 (1.07-1.36)**** |
| HRs (95% CIs) were derived from Cox proportional hazard models.  Model 1: sex and age  Model 2: model 1+ + high alcohol intake, BMI, lipid-lowering medication , anti-hypertensive medication, and family history of diabetes  Model 3a: model 2+ HDL-C; Model 3b: model 2+ HDL size  Model 4a: model 3a + HOMA‐β; Model 4b: model 3b + HOMA‐β  Model 5a: model 4a + HOMA‐ IR; Model 5b: model 4b + HOMA‐ IR  *P<0.05; **P<0.01; ***P< 0.001.  TRLP: triglyceride rich lipoprotein particles; HOMA-IR: Homeostatic model assessment of insulin resistance; HOMA‐β : Homeostatic model assessment of β-cell function; BMI: Body mass index; HDL-C: High-density lipoprotein cholesterol. | | | | | | | |

| **Table S7**. Association between LDL subfractions and risk of T2D in 4818 people without diabetes at baseline | | | | | |
| --- | --- | --- | --- | --- | --- |
| HR (95% CI) per SD-increase,  *P*-values | | | | | |
| LDL subfractions | Total LDLP | Large LDLP | Medium LDLP | Small LDLP | LDL size |
| Crude | **1.46 (1.28-1.66)***** | **0.68 (0.63-0.73)***** | **0.84 (0.75-0.94)**** | **1.53 (1.25-1.87)***** | **0.62 (0.57-0.69)***** |
| Model 1 | **1.34 (1.17-1.53)***** | **0.69 (0.64-0.75)***** | **0.82 (0.73-0.91)***** | **1.42 (1.18-1.71)***** | **0.64 (0.58-0.71)***** |
| Model 2 | **1.25 (1.09-1.42)**** | **0.77 (0.70-0.84)***** | 0.90 (0.81-1.01) | **1.17 (1.01-1.37)*** | **0.75 (0.67-0.84)***** |
| Model 3a | **1.16 (1.01-1.32)*** | **0.80 (0.73-0.87)***** | **0.89 (0.79-0.99)*** | 1.11 (0.96-1.28) | **0.80 (0.71-0.91)***** |
| Model 3b | 1.07 (0.93-1.22) | **0.83 (0.76-0.91)***** | **0.88 (0.78-0.98)*** | 1.08 (0.94-1.24) | **0.86 (0.75-0.97)*** |
| Model 4a | **1.18 (1.03-1.35)*** | **0.77 (0.71-0.85)***** | **0.87 (0.78-0.98)*** | 1.14 (0.98-1.32) | **0.78 (0.69-0.89)***** |
| Model 4b | 1.07 (0.93-1.23) | **0.81 (0.74-0.89)***** | **0.85 (0.77-0.95)**** | 1.10 (0.96-1.26) | **0.84 (0.74-0.96)**** |
| Model 5a | 1.11 (0.98-1.27) | **0.84 (0.76-0.92)***** | 0.91 (0.82-1.02) | 1.08 (0.94-1.23) | **0.84 (0.74-0.95)**** |
| Model 5b | 1.05 (0.92-1.21) | **0.86 (0.78-0.94)**** | **0.89 (0.80-0.99)*** | 1.06 (0.93-1.21) | **0.87 (0.77-0.99)*** |
| HRs (95% CIs) were derived from Cox proportional hazard models.  Model 1: sex and age  Model 2: model 1+ high alcohol intake, BMI, lipid-lowering medication , anti-hypertensive medication, and family history of diabetes  Model 3a: model 2+ HDL-C; Model 3b: model 2+ HDL size  Model 4a: model 3a + HOMA‐β; Model 4b: model 3b + HOMA‐β  Model 5a: model 4a + HOMA‐ IR; Model 5b: model 4b + HOMA‐ IR  *P<0.05; **P<0.01; ***P< 0.001.  LDLP: low density lipoprotein particles; HOMA-IR: Homeostatic model assessment of insulin resistance; HOMA‐β : Homeostatic model assessment of β-cell function; BMI: Body mass index; HDL-C: High-density lipoprotein cholesterol. | | | | | |

| **Table S8**. Association between large TRLP, TRL size , and small LDLP and risk of T2D in 203 subjects with high alcohol intake and 4615 subjects with no high alcohol intake | | | | | | | | | |
| --- | --- | --- | --- | --- | --- | --- | --- | --- | --- |
| HR (95% CI) per SD-increase,  *P*-values | | | | | | | | | |
|  |  | **Large TRLP** | | | |  | | **TRL size** | **Small LDLP** |
| **High alcohol intake (incident T2D: n=18)** | | | | | | | |  |  |
| Crude | | |  | | 1.25 (0.69-2.25) | |  | 1.14 (0.79-1.65) | 0.89 (0.60-1.33) |
| Model 1 | | |  | | 1.24 (0.69-2.21) | |  | 1.17 (0.81-1.69) | 0.88 (0.59-1.30) |
| Model 2 | | |  | | 0.97 (0.52-1.78) | |  | 1.03 (0.69-1.55) | 0.80 (0.55-1.16) |
| Model 3a | | |  | | 0.82 (0.44-1.53) | |  | 0.99 (0.64-1.53) | 0.78 (0.54-1.11) |
| Model 3b | | |  | | 0.84 (0.43-1.62) | |  | 1.01 (0.66-1.53) | 0.79 (0.55-1.14) |
| Model 4a | | |  | | 0.88 (0.48-1.62) | |  | 0.98 (0.64-1.49) | 0.74 (0.52-1.07) |
| Model 4b | | |  | | 0.92 (0.47-1.82) | |  | 1.02 (0.68-1.53) | 0.75 (0.51-1.10) |
| Model 5a | | |  | | 0.83 (0.44-1.56) | |  | 1.00 (0.66-1.51) | 0.76 (0.51-1.12) |
| Model 5b | | |  | | 0.85 (0.42-1.70) | |  | 1.04 (0.69-1.58) | 0.75 (0.50-1.12) |
|  | | |  |  | |  | |  |  |
| **No high alcohol intake (incident T2D: n=245)** | | | | | | | | | |
| Crude | | |  | | **2.34 (1.98-2.75)***** | |  | **1.72 (1.56-1.90)***** | **1.60 (1.29-1.98)***** |
| Model 1 | | |  | | **2.22 (1.88-2.62)***** | |  | **1.70 (1.54-1.88)***** | **1.48 (1.21-1.80)***** |
| Model 2 | | |  | | **1.77 (1.49-2.10)***** | |  | **1.49 (1.33-1.66)***** | **1.18 (1.00-1.39)*** |
| Model 3a | | |  | | **1.67 (1.41-1.99)***** | |  | **1.46 (1.31-1.63)***** | 1.11 (0.95-1.30) |
| Model 3b | | |  | | **1.55 (1.29-1.86)***** | |  | **1.38 (1.23-1.56)***** | 1.10 (0.94-1.25) |
| Model 4a | | |  | | **1.85 (1.55-2.20)***** | |  | **1.55 (1.39-1.72)***** | 1.15 (0.98-1.34) |
| Model 4b | | |  | | **1.68 (1.40-2.01)***** | |  | **1.44 (1.29-1.62)***** | 1.10 (0.95-1.27) |
| Model 5a | | |  | | **1.46 (1.23-1.74)***** | |  | **1.27 (1.12-1.43)***** | 1.08 (0.94-1.25) |
| Model 5b | | |  | | **1.39 (1.16-1.67)**** | |  | **1.22 (1.08-1.38)**** | 1.06 (0.93-1.23) |
| HRs (95% CIs) were derived from Cox proportional hazard models.  Model 1: sex and age  Model 2: model 1+ BMI, anti-hypertensive medication, lipid-lowering medication, and family history of diabetes  Model 3a: model 2+ HDL-C; Model 3b: model 2+ HDL size  Model 4a: model 3a + HOMA‐β; Model 4b: model 3b + HOMA‐β  Model 5a: model 4a + HOMA‐ IR; Model 5b: model 4b + HOMA‐ IR  *P<0.05; **P<0.01; ***P< 0.001.  LDLP: low density lipoprotein particles; TRLP: triglyceride rich lipoprotein particles; HOMA-IR: Homeostatic model assessment of insulin resistance; HOMA‐β : Homeostatic model assessment of β-cell function; BMI: Body mass index; HDL-C: High-density lipoprotein cholesterol. | | | | | | | | | |
